# Giant urinary bladder calculi in a 60-year-old man: a case report

**DOI:** 10.11604/pamj.2022.41.78.33131

**Published:** 2022-01-27

**Authors:** Faisal Ahmed, Qasem Alyhari, Saleh Al-wageeh, Fawaz Mohammed

**Affiliations:** 1 Urology Research Center, Al-Thora General Hospital, Department of Urology, School of Medicine, Ibb University of Medical Science, Ibb, Yemen,; 2 Department of General Surgery, School of Medicine, Ibb University of Medical Science, Ibb, Yemen,; 3 Department of Orthopedy, School of Medicine, Ibb University of Medical Science, Ibb, Yemen

**Keywords:** Bladder stone, cystolithotomy, giant, case report

## Abstract

Urinary bladder calculi comprise 5% of all urinary tract calculi. Giant bladder calculi are defined as a stone more than 100g in weight. However, giant bladder calculus weighted more than 500g is rare in current practice. We present a 60-year-old man who presented with dysuria, difficulty in urination, and suprapubic pain started four years ago. The plain radiology image showed big intravesical caliculi measured about 10x9cm. The calculi was removed via open cystolithotomy without postoperative complication. The caliculi weighed 750g. In conclusion, the main goal of treatment is to remove the calculi and relieve the accompanying symptoms.

## Introduction

Giant urinary bladder calculi are defined as a stone more than 100g in weight, a rare condition in current practice [1]. Urinary tract infection, urethral stricture, benign prostatic hyperplasia, and intravesical foreign body encrustation are the most common causes of urinary bladder calculi formation [2]. Bladder calculi present in various ways, ranging from entirely asymptomatic to dysuria, suprapubic pain, hematuria, and urine retention [3]. A few published reports regarding giant bladder stones weighed more than 500g [4]. Here we present a case of giant bladder calculi in a 60-year-old man. The manifestations, diagnosis, and treatment are discussed.

## Patient and observation

**Patient information:** a 60-year-old Yemeni man, illiterate, presented to our urology department at Althora general hospital in September 2021 with a chief complaint of dysuria, difficulty in urination, and suprapubic pain started four years ago. There is no history of abdominal trauma, neurological disease, or urologic disease such as urinary tract stones.

**Clinical findings:** regarding the physical examination, the patient had severe, nonradiated suprapubic pain associated with mild tenderness and detectable enlarged and nonmobile suprapubic mass in palpation.

**Figure 1:**
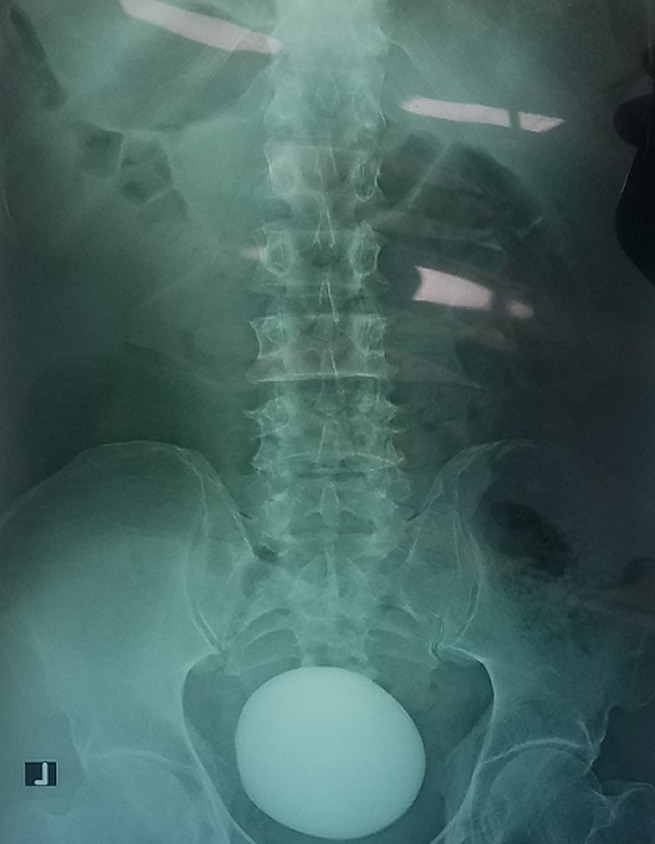
giant bladder stone in plain radiography

**Diagnostic assessment:** blood tests revealed a total white blood cell count of 12x10^3^/ml with moderate leukocytosis and hemoglobin: 14.4g/dl, blood urea nitrogen: 14mg/dl, and creatinine: 1.1mg/dl. Urine analysis showed microscopic hematuria (15-20 RBCs/HPF) and many pass cells (20 WBCs/HPF). The abdominal X-ray showed giant intravesical calculus measured 10x9cm (Figure 1). The ultrasonography showed moderate hydronephrosis in both kidneys, mild bladder wall thickness, giant intravesical calculi, and mild prostate enlargement.

**Figure 2:**
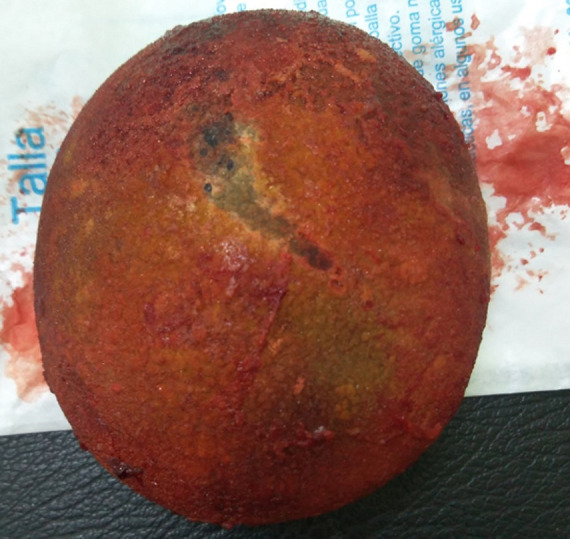
a 10x9cm bladder stone removed by open cystolithotomy

**Therapeutic interventions:** the antibiotic (Ceftriaxone g every 12hours for three days) was started to control urinary tract infection. Then, the patient was admitted to elective surgery. Via spinal anesthesia in the supine position, open cystolithotomy was made with a lower umbilical midline incision, the bladder was opened, and large calculus adhered to the bladder wall and measured about 10x9x5cm was observed and removed. Three ways urethral catheter was inserted, and the bladder and skin were finally closed. The calculi weighed about 750g (Figure 2).

**Follow-up and outcome:** postoperative hospital course was uneventful. The patient was discharged with an oral antibiotic on the third postoperative day, and the catheter was removed on the seventh postoperative day. Within three months of follow-up, the patient remained symptom-free.

**Patient perspective:** the patient was happy with the successful outcome of the surgery.

**Informed consent:** written informed consent was obtained from the patient for participation in our study.

## Discussion

Giant urinary bladder calculi is a rare condition and defined stone more than 100g in weight [1]. It is more frequent in males than females [2]. Giant urinary bladder calculi most often occur with other pathologic bladder conditions such as urinary retention, urethral stricture, urinary tract infection, prolonged catheterization, neurogenic bladder, and the existence of the foreign body [5].

The pathophysiology of giant bladder calculi can develop from a nidus of infected material or single ureteric calculus with a progressive layer-wise accumulation of calcified matrix associated with factors causing urinary stasis such as bladder outlet obstruction [2]. Factors such as low educational level, low socioeconomic, dry climate, dietary habits, and high exposure to the sun are associated with a high incidence of urinary tract calculi [6]. Our patient was illiterate, worked outdoor with high exposure to the sun.

The clinical manifestations of giant bladder calculi range from entirely asymptomatic to acute urinary retention [4]. Our patient was suffered from dysuria, difficulty in urination, and suprapubic pain. The vast majority of bladder calculi are radiopaque and can be identified with a plain radiograph. Other useful radiologic images are ultrasonography, computed tomography scan, magnetic resonance imaging, and intravenous pyelography [7,8].

There are several surgical options for bladder stone, including open cystolithotomy, extracorporeal fragmentation, percutaneous endoscopic cystolitholapexy, and cystolitholapexy. Open cystolithotomy has been recommended as the best treatment option for large bladder calculi [3,4]. Similarly, our patient was undergoing open cystolithotomy without complication.

Few recently published reports with giant bladder calculi weighted more than 500g such as Ma and associations who reported a bladder calculi with 1048g in weight [4], Shrestha *et al*. reported a bladder calculi with 950g in weight [9], Nugroho and associations reported a bladder calculi with 832g in weight [3], and the recent report by Pattiiha et al. reported a bladder calculi with 500g in weight [6]. Similarly, our patient had giant bladder calculus that weighed 750g.

## Conclusion

Giant urinary bladder calculi with more than 500g in weight are rare in current urologic practice. The radiologic diagnostic methods of bladder calculi include ultrasonography, plain radiography, and computed tomography scan. Open cystolithotomy is our patient's best surgical option in giant bladder calculi.
